# Evaluation of the impact of guideline communication from the Centers for Disease Control and Prevention and the Centers for Medicare and Medicaid Services among US healthcare providers: COVID‐19 prevention counselling guidance

**DOI:** 10.1002/nop2.1862

**Published:** 2023-05-30

**Authors:** Melanie M. Taylor, Arkaprava Deb, Bernita Frazier, James Reiss Lueken, Mansi Das, Joanna Molke, Erin Fitzgerald, Tom Ullian, Ridhu Nair, Marion Couch, Caitlin Turbyfill, Libby Horter, Cecilia Joshi, Nickolas DeLuca

**Affiliations:** ^1^ COVID‐19 Response, State, Tribal, Local, and Territorial Support Task Force, Contact Tracing and Innovation Section Centers for Disease Control and Prevention Atlanta Georgia USA; ^2^ Hospital and Ambulatory Policy Group Centers for Medicare and Medicaid Services Woodlawn Maryland USA; ^3^ Office of the Assistant Director of Communications Centers for Disease Control and Prevention Atlanta Georgia USA; ^4^ Sermo New York New York USA; ^5^ RNsights Braintree Massachusetts USA

**Keywords:** contact tracing, COVID‐19, Medicaid, Medicare, provider billing, provider counselling, reimbursement

## Abstract

**Aim:**

To evaluate healthcare provider awareness and uptake of the Centers for Medicare & Medicaid Services (CMS) billing for coronavirus disease 2019 (COVID‐19) prevention counselling and the delivery of prevention counselling to patients awaiting severe acute respiratory syndrome coronavirus 2 test results.

**Design:**

Cross sectional survey of US‐based healthcare providers in February 2021.

**Methods:**

Analysis of associations with healthcare provider‐reported awareness of CMS prevention counselling guidance and billing with provider type, specialty, and work setting.

**Results:**

A total of 1919 healthcare providers responded to the survey. Overall, 38% (726/1919) of providers reported awareness of available CMS reimbursement for COVID‐19 patient counselling and 29% (465/1614) of CMS billing‐eligible providers reported billing for this counselling. Among physicians, those aware of CMS guidance were significantly more likely to bill (58%) versus those unaware (10%). Among RNSights respondents eligible for CMS billing (*n* = 114), 31% of those aware of the guidance reported billing as compared to 0% of those not aware.

## 1 BACKGROUND

In June 2020, the Centers for Disease Control and Prevention (CDC) worked with the Centers for Medicare & Medicaid Services (CMS) to create and disseminate coronavirus disease 2019 (COVID‐19) prevention counselling recommendations for healthcare providers to provide to patients awaiting severe acute respiratory syndrome coronavirus 2 (SARS‐CoV‐2) test results Centers for Medicare & Medicaid Services (2020a). CDC and CMS surmised that a public health benefit would accrue if healthcare providers counselled patients at the time of testing about positive COVID‐19 test results and what to do while awaiting those results. CDC and CMS developed prevention counselling messages including topics such as self‐isolation and quarantine, notifying household contacts, and encouraging cooperation with public health COVID‐19 case investigation and contact tracing (Centers for Medicare & Medicaid Services, 2020b. Counselling check list; Centers for Medicare & Medicaid Services, 2020c. Provider Q&A; Centers for Medicare & Medicaid Services, 2020d. Talking points for providers; Centers for Disease Control and Prevention, 2019a. Contact tracing; Centers for Disease Control and Prevention, 2019b. Key steps to take while waiting for your COVID‐19 test result). CMS provided guidance to physicians on including time spent counselling patients in selecting the appropriate code level of the visit using existing evaluation and management (E&M) codes for Medicare and Medicaid reimbursement for COVID‐19‐related counselling time.

Billing‐eligible healthcare providers can be reimbursed by CMS for services that include COVID‐19 counselling using existing current procedural terminology (CPT®) E&M codes and payment policies. This counselling and billing initiative by CMS and CDC was intended to encourage medical providers to provide prevention counselling that included isolation and quarantine at the time of SARS‐CoV‐2 testing. The CDC/CMS initiative was announced and rolled out by CMS in July 2020. Guidance was delivered through CMS' Medicare Learning Network distribution lists, CMS and CDC websites, and direct communication with Medicare's local contractors. These communications are usually amplified by multiple platforms and listservs from physician specialty societies (such as the American Medical Association). The primary objective of this study was to capture information about provider awareness and delivery of CDC‐ and CMS‐recommended patient counselling messages regarding COVID‐19. The secondary objective was to determine if providers ordering SARS‐CoV‐2 testing for patients are billing for COVID‐19 counselling services provided.

## 2 METHODS

### 2.1 Survey development

CDC led the development of a 10‐item survey based on CMS and CDC COVID‐19 counselling and billing information in November 2020 (Appendix A). We gathered feedback from CDC subject matter experts (SMEs) with knowledge of the delivery of counselling services as part of public health outreach about the appropriateness of survey items. SMEs included physicians, public health analysts, disease investigators, case managers, and contact tracers. We also asked them to take the survey to determine the time needed to complete it and to assess the wording of the items to ensure they were easy to understand and accurately captured the counselling topics. The estimated time to complete the survey was 3–5 min.

### 2.2 Survey distribution

In February 2021, CDC partnered with the medical provider membership and resource platforms Sermo (https://www.sermo.com/about/) and RNsights (https://rnsights.com/about‐us/) to distribute the CDC survey to a convenience sample of United States‐based, licensed, practicing healthcare providers. The aim of the survey was to assess provider awareness and delivery of CDC‐ and CMS‐recommended COVID‐19 counselling and associated self‐reported billing practices. In addition, providers were asked about their general knowledge of the CMS/CDC recommendations on counselling for patients testing for COVID‐19, the sources from which they were made aware of counselling recommendations, and the opportunity to bill for this counselling activity. There was no associated compensation or incentive for participation.

### 2.3 Survey population and distribution

Sermo is a peer‐to‐peer social platform and healthcare research company membership site that engages with 1.3 million healthcare providers across 150 countries; over 300,000 of these are U.S.‐based physicians. RNsights is an online platform that hosts a membership of more than 700,000 U.S.‐based nurse practitioners (NPs), physician assistants (PAs), and registered nurses and provides engagement opportunities with its members.

Survey distribution was restricted to U.S.‐based providers. Inclusion criteria for receipt of the survey were: patient care providers within general practice and medical specialties, including physicians, NPs, PAs, and nurses. The following specialties were excluded from survey distribution: hospital administrators, radiologists, ophthalmologists, dermatologists, pathologists, and laboratorians. All responding healthcare providers were included in frequency analyses of awareness and delivery of COVID‐19 counselling. Only CMS billing‐eligible healthcare providers (physicians, physicians' assistants, and NPs) were included in the frequency analyses of billing.

The survey administered through Sermo was disseminated via email to 5442 physicians on 1 February 2021 and closed on 1 March 2021 (4 weeks). The Sermo survey response was capped at 1500 respondents. The survey administered through RNsights was launched via email link to 440,000 recipients on 3 February 2021 and closed on March 5 (4 weeks). Two email reminders were sent during the survey period.

These survey intervals and cap of 1500 for Sermo reflected the agreement with CDC for a no‐cost survey delivery and data collection. The results were two convenience samples of self‐selected individuals who volunteered to participate in the survey.

### 2.4 Data analysis

Descriptive statistics on provider type, specialty, and work setting were performed using SAS (V9.4). The relationship between provider awareness and billing was explored using either the chi‐square or Fisher's exact test, as appropriate. Prevalence ratios (PR) were calculated using robust Poisson regression to estimate the probability of billing or providing any counselling based on the location of the worksite. Regression results were adjusted for hospital place of work to discriminate between inpatient and non‐inpatient places of counselling, given the time and resource differential between inpatient and outpatient services.

### 2.5 Ethics approval

This activity underwent CDC ethical board review, received approval (# 0900f3eb81c5e94b), and was conducted consistent with applicable federal law and CDC policy. (E.g., 45 C.F.R. part 46, 21 C.F.R. part 56; 42 U.S.C. §241(d); 5 U.S.C. §552a; 44 U.S.C. §3501 et seq.).

## 3 RESULTS

A total of 1500 physician respondents from all 50 states participated in the survey delivered by Sermo; 61% of providers reported working in a doctor's office or outpatient clinic, and 31% reported working in a hospital setting (Table 1). Of physician respondents, 42% (629/1500) reported awareness of CMS reimbursement for patient counselling at the time of SARS‐CoV‐2 testing, and 30% (453/1500) self‐reported as having billed for these services (Table 2). Of the 629 Sermo respondents who were aware, 365 (58%) billed and 264 (42%) did not bill; and of the 871 Sermo respondents who were not aware, 88 (10%) billed and 783 (90%) did not bill (*p* < 0.001). Reported sources of the CMS/CDC information included: from a colleague (21%, *n* = 315); CDC Internet site (19%, *n* = 279); professional association (18%, *n* = 277); and general Internet search (16%, *n* = 237) (Table 2).

**TABLE 1:** Description of healthcare provider respondents of CDC survey, Sermo, and RNsights (*N* = 1919).

	Sermo	RNsights	Overall
	*N* = 1500	*N* = 419	*N* = 1919
Provider type, *N* (%)			
Nurse	^a^	305 (73)	305 (16)
Nurse practitioner	^a^	92 (22)	92 (5)
Physician assistant	^a^	22 (5)	22 (1)
Physician	1500 (100)	^a^	1500 (78)
Specialty, *N* (%)			
Family or general practice	318 (21)	37 (9)	355 (19)
Internal medicine	186 (12)	15 (4)	201 (10)
Paediatrics	154 (10)	20 (5)	174 (9)
Obstetrics & gynaecology	99 (7)	6 (1)	105 (5)
Cardiology	87 (6)	13 (3)	100 (5)
Emergency medicine or emergency services	72 (5)	21 (5)	93 (5)
Pulmonology/respiratory medicine	52 (3)	0	52 (3)
General surgery	55 (4)	17 (4)	72 (4)
Intensive care/critical care medicine	18 (1)	23 (5)	41 (2)
Oncology	64 (4)	14 (3)	78 (4)
Anaesthesiology	49 (3)	3 (1)	52 (3)
Psychiatry	6 (<1)	15 (4)	21 (1)
Otolaryngology (ORL/ENT)	22 (2)	2 (1)	24 (1)
Neurology	45 (3)	4 (1)	49 (3)
Endocrinology	42 (3)	^a^	42 (2)
Dermatology	37 (3)	^a^	37 (2)
Gastroenterology	31 (2)	2 (1)	33 (2)
Allergy & immunology	30 (2)	^a^	30 (2)
Rheumatology	26 (2)	^a^	26 (1)
Nephrology	23 (2)	^a^	23 (1)
Infectious diseases	22 (2)	^a^	22 (1)
Urology	19 (1)	2 (1)	21 (1)
Physical medicine & rehabilitation (physiatry)	16 (1)	17 (4)	33 (2)
Pain medicine	11 (0.7)	2 (1)	13 (2)
Hospital medicine	6 (<1)	3 (1)	9 (1)
School nurse	^a^	84 (20)	84 (4)
Nurse educator	^a^	15 (4)	15 (1)
Home health, long term or ambulatory care	^a^	25 (6)	25 (1)
Community health	^a^	7 (2)	7 (<1)
Other	10 (1)	63 (15)	73 (4)
Work setting, *N* (%)^b^			
Outpatient clinic	263 (18)	84 (20)	347 (18)
Doctors office	658 (44)	49 (12)	707 (37)
Federally qualified health centre	29 (2)	9 (2)	38 (2)
Hospital	462 (31)	135 (32)	597 (31)
Non‐healthcare	7 (1)	34 (8)	41 (2)
Pharmacy	4 (<1)	0	4 (0)
Rural health centre	9 (1)	7 (2)	16 (1)
Urgent care	34 (2)	7 (2)	41 (2)
None listed/missing	30 (2)	35 (8)	35 (8)
Other	0	120 (29)	120 (6)
Local health department	4 (<1)	0	4 (<1)

^a^ Not applicable.

^b^ RNsights survey allowed for multiple responses for worksite.

**TABLE 2:** Awareness and uptake of CDC/CMS reimbursement on COVID‐19 counselling and sources of information on CMS/CDC counselling messages.

Survey question	Sermo *N* = 1500	RNsights *N* = 419	Overall *N* = 1919
Familiar with CMS/CDC information released in July 2020	629 (42)	97 (23)	726 (38)
Billed medicare with E/M codes for COVID‐19 counseling^a^	453 (30)	12 (11)	465 (29)
Received or became aware of the CMS/CDC information from^b^			
CDC Internet site	279 (19)	47 (11)	326 (17)
CMS Internet site	158 (11)	15 (4)	173 (9)
State or local health department Internet site	206 (14)	27 (6)	233 (12)
General Internet search	237 (16)	31 (7)	268 (14)
Professional association	277 (18)	32 (8)	309 (16)
Subscription to a professional education platform	133 (9)	6 (1)	139 (7)
A colleague	315 (21)	30 (7)	345 (18)
A listserv	94 (6)	6 (1)	100 (5)
Other	36 (2)	15 (4)	51 (3)
Not aware	675 (45)	278 (66)	953 (50)

^a^ Limited to 1614 providers (Sermo, *n* = 1500; RNsights, *n* = 114) eligible to bill Medicare/Medicaid; 305 RNs excluded.

^b^ Not mutually exclusive.

Abbreviations: CDC, Centers for Disease Control and Prevention; CMS, Centers for Medicare & Medicaid Services; COVID‐2019, coronavirus disease 2019.

There were 423 respondents to the RNsights survey. Four respondents noted no connection to SARS‐CoV‐2 testing or COVID‐19 counselling, so analyses were performed on 419 records. Respondents included 305 nurses, 92 NPs, and 22 physicians' assistants (Table 1). Among this sample, 23% (97/419) reported awareness of available CMS reimbursement for patient counselling at the time of SARS‐CoV‐2 testing. There were 114 respondents who were eligible to bill Medicare/Medicaid. Among them, 34% (39/114) were aware of reimbursement, and 11% (12/114) of these providers self‐reported as having billed for COVID‐19 counselling. For the RNsights respondents eligible to bill, of the 39 who were aware, 12 (31%) billed and 27 (69%) did not bill. Of the 75 who were unaware, none billed (*p* < 0.001). Reported sources of the CMS/CDC information included: CDC Internet site (11%, *n* = 47); a professional association (8%, *n* = 32); a colleague (7%, *n* = 30), and general Internet search (7%, *n* = 31) (Table 2).

**FIGURE 1:**
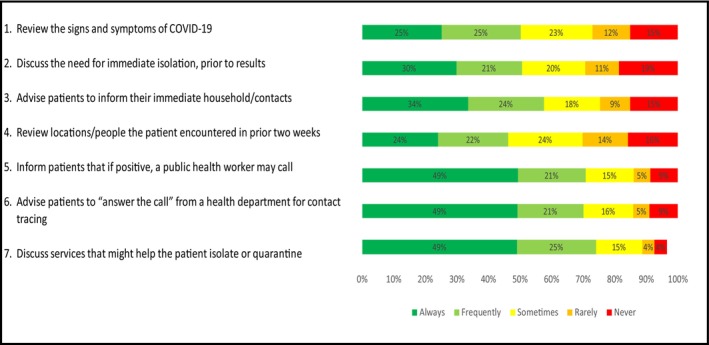
Healthcare providers' self‐reported content of counselling for patients awaiting coronavirus disease 2019 (COVID‐19) test results (*N* = 1919).

Among the entire sample, (*N* = 1919), self‐reporting of consistent COVID‐19 counselling (responses ‘always’ or ‘frequently’) was reported for the recommended counselling topics regardless of awareness of billing and reimbursement: (1) review the signs and symptoms of COVID‐19 (74%); (2) discuss the need for immediate isolation, even before test results are available (70%); (3) advise patients to inform their immediate household/contacts that they may wish to be tested and quarantined (70%); (4) review locations and people the patient may have been in contact with in the past 2 weeks (46%); (5) inform patients that if they test positive for COVID‐19, they will likely be contacted by a public health worker (58%); (6) advise patients to ‘answer the call’ from a public health worker to provide a list of people they have been with for contact tracing purposes (51%); and (7) discuss services that might help the patient to successfully isolate or quarantine at home (50%) (Figure 1).

Among physicians responding to the Sermo survey, those working in hospitals were less likely to report providing any counselling as compared to providers working in outpatient settings (PR 0.78/0.86 = 0.91, 95% CI 0.86, 0.96). Billing for COVID‐19 counselling by hospital providers was reported more often as compared to physicians in outpatient worksites, although these results were not significant (PR 33.55/29.37 = 1.14, 95% CI 0.97, 1.34).

## 4 DISCUSSION

Results from this convenience sample of nearly 2000 healthcare providers in the United States demonstrate awareness of, delivery of, and reimbursement for CDC‐ and CMS‐recommended COVID‐19 counselling prevention recommendations. Healthcare providers who were aware of the CMS guidance were more likely to bill for Medicare and Medicaid reimbursement for COVID‐19‐related counselling time. Among healthcare providers aware of recommendations and reimbursement, CDC and CMS websites were reported as sources of this information. Counselling regarding signs and symptoms, isolation, and notifying household members was reported more often than counselling regarding health department‐related contact tracing activities. Among physicians, those working in hospital settings were less likely to provide COVID‐19 prevention counselling as compared to those working in outpatient settings.

CDC, CMS, and other healthcare partners may consider these survey findings as opportunities to increase provider awareness of COVID‐19 prevention counselling recommendations and CMS reimbursement. Additional evaluation could help determine effective methods and platforms for use to rapidly deliver such information to providers that can then be consistently relayed to patients. Healthcare providers deliver prevention counselling for other communicable, acute and chronic conditions, and this experience can be considered when developing evaluation methods (Kamb et al., 1996; Omura et al., 2018; Petersen et al., 2007; Searight, 2018; Shen et al., 2019; Wolff et al., 2010). Counselling to address mental health concerns such as fear and anxiety associated with COVID‐19 has been identified as an important and unmet need (Costanza et al., 2021; Nagata et al., 2022). The relatively new advance care planning CPT code and its utilization are examples of how CMS has adopted a payment policy and providers have responded with increased reporting of counselling services. Advance care planning (CPT code 99497) services largely consist of counselling and care planning related to specific topics of advanced disease management. The utilization of this CPT code has increased from over 600,000 Medicare claims in 2016 to over 1,800,000 claims by 2019 (Palmer et al., 2021). Similarly, knowledge of the CDC's and CMS's recommendations may enhance a provider's ability to deliver COVID‐19 prevention counselling to patients. As of 13 December 2021, there were 20,211 unique web visits to the CMS announcement for COVID‐19 counselling reimbursement, 5, 125 web visits to the CMS provider COVID‐19 counselling checklist, and 1003 unique visits to the CMS webpage for provider Q & A on CMS reimbursement for COVID‐19 counselling. CDC promotes public‐private partnerships that include healthcare provider outreach, virtual education sessions and communication activities (Centers for Disease Control and Prevention, 2021c. Public‐private partnerships and CDC; Centers for Disease Control and Prevention, 2021a. CDC's COVID‐19 partner calls). These have been used to improve healthcare provider and partner awareness of various aspects of COVID‐19 care and prevention, including case investigation, contact tracing, and vaccination.

Working in partnership with public health personnel, providers may influence the participation of people with COVID‐19 and their contacts with public health investigations and contact tracing. CDC modelling estimates suggest that prompt isolation of people with COVID‐19 can significantly reduce transmission (Centers for Disease Control and Prevention, 2021b. Prioritizing COVID‐19 contact tracing mathematical modelling methods and findings). However, the limited willingness of patients to participate in interviews and provide information on contacts reduces the prevention benefit of contact tracing (Lash et al., 2021). Healthcare providers who provide concurrent COVID‐19 counselling during medical encounters that include SARS‐CoV‐2 testing may be able to influence patients to engage with health department‐delivered case investigation and contact tracing and comply with timely prevention efforts, including isolation and quarantine, to reduce transmission.

### 4.1 Strength and limitations

The results from this survey identify opportunities to educate healthcare providers and administrators on public health prevention activities and associated counselling for patients. The results of these self‐selected convenience surveys should be interpreted with caution. Sample selection limits the generalization of results to a broader group of US‐based healthcare providers testing for SARS‐CoV‐2. The response rate was low for the RNsights survey, and few billing‐eligible providers participated. Some responding providers, such as those working in surgical fields, may be providing pre‐procedure screening testing and not diagnostic SARS‐CoV‐2 testing and thus not taking the opportunity to provide counselling. The survey did not distinguish providers who were rarely involved in testing patients who may have chosen ‘rarely’ or ‘never’ for counselling. Nurses were included in this survey based on their role in providing prevention counselling to patients; however, they may not necessarily be expected to have awareness of CMS billing and reimbursement. This would not be expected to impact their willingness to provide counselling. Hospital providers may not be able to counsel critically ill patients at the time of testing, thus explaining the lower coverage of consistent counselling. Demographics of respondents such as provider age, gender, and practice in rural versus urban settings could have affected knowledge and delivery of counselling, but these data were not collected. The specific types of colleagues that were chosen as reported sources of information on CMS counselling and billing were not collected. Billing information was self‐reported and not verified objectively. Finally, some eligible providers may not have billed CMS because of the patient population they serve, which may not include Medicare/Medicaid enrolled patients.

Healthcare providers can play a key role not only in identifying and treating COVID‐19 but also in providing critical information to patients that can slow the spread of SARS‐CoV‐2. This evaluation of provider delivery of COVID‐19 counselling identified opportunities to improve the knowledge and consistent delivery of CDC‐ and CMS‐recommended prevention messages related to isolation and contact tracing. Providers also have the opportunity to deliver other key prevention strategies, such as the importance of obtaining a COVID‐19 vaccine. They may be further encouraged to perform COVID‐19 counselling consistent with recommendations if they are aware of and use CMS billing and reimbursement options. This may be especially relevant at this stage in the COVID‐19 pandemic, where there is a need for healthcare providers to emphasize the benefits of vaccination (Centers for Medicare and Medicaid Services., 2021. CMS COVID‐19 vaccine page for providers). Counselling of Medicaid clients is especially important given the association of lower socioeconomic status and other demographic factors commonly found in this population with higher risks of COVID‐19 morbidity (Romano et al., 2021; Smith et al., 2021).

## 5 CONCLUSION

Activities such as increased communication and education to improve provider awareness the and delivery of counselling may result in patient behaviours that prevent SARS‐CoV‐2 transmission, reduce the burden of COVID‐19, and save lives. Ongoing efforts to guide and support healthcare providers in delivering prevention counselling might help stem the transmission of SARS‐CoV‐2 and other communicable infections.

## AUTHOR CONTRIBUTIONS

Melanie M. Taylor, Nickolas DeLuca, Bernita Frazier, and James Reiss Lueken designed the study, Bernita Frazier, James Reiss Lueken, Mansi Das, Erin Fitzgerald, Tom Ullian, and Ridhu Nair implemented the survey, Caitlin Turbyfill and Libby Horter performed the analysis of data. Melanie M. Taylor, Nickolas DeLuca, Caitlin Turbyfill, and Libby Horter wrote the manuscript. All authors contributed to the review and finalization of the draft.

## FUNDING INFORMATION

None.

## CONFLICT OF INTEREST STATEMENT

The authors declare no conflicts of interest.

## DISCLAIMER

The findings and conclusions in this report are those of the authors and do not necessarily represent the official position of the Centers for Disease Control and Prevention.

## Data Availability

The data that support the findings of this study are available from the corresponding author upon reasonable request.

## A.1 SURVEY

Opening: The CDC is teaming up with Sermo and RNSights to evaluate healthcare provider awareness and uptake of Centers for Medicare and Medicaid (CMS) and Centers for Disease Control and Prevention (CDC) information released in July 2020 about Medicare reimbursement for COVID‐19 counselling for patients at the time of SARS CoV‐2 testing. Our goal is to have approximately 1000 U.S. HCPs take part in this 3–5‐min survey. There is no compensation for your time, but your valuable input will help the CDC inform future COVID‐19 counselling guidance so we can effectively combat this disease together. Thank you in advance for your participation.

1. Are you familiar with CMS/CDC information released in July 2020 that describes reimbursement from Medicare that is available to healthcare providers who counsel patients at the time of SARS‐CoV‐2 testing? This information can be found on the CMS website. Select One.
  - Yes
  - No
2. Please identify how you received or became aware of the CMS/CDC information for providers counselling patients on COVID‐19. Select all that apply.

- Downloaded from CDC Internet site
- Downloaded from CMS Internet site
- Downloaded from state or local health department Internet site
- General Internet search
- Received guidance from my professional association
- Received guidance from my subscription to a professional education platform
- Received guidance from a colleague
- Received guidance shared on a listserv
- Other (please specify.) _________________
- I am not aware of the information

3 Have you billed Medicare with Evaluation and Management Services (E/M) codes for COVID‐19 counselling for patients receiving a SARS‐CoV‐2 test? Select One.

- Yes
- No

Please indicate how often you deliver COVID‐19 counselling for patients receiving a COVID‐19 test using topics listed below.

**Table:**

CMS/CDC recommended COVID‐19 counselling topics	Always	Frequently	Sometimes	Rarely	Never
4 Review the signs and symptoms of COVID‐19					
5 Discuss the need for immediate isolation, even before test results are available					
6 Advise patients to inform their immediate household/contacts to get tested and quarantine					
7 Review locations and people the patient may have been in contact within the past 2 weeks					
8 Inform patients that if positive, they will likely be contacted by a public health worker					
9 Advise patients to ‘answer the call’ from a public health worker to provide a list of people they have been with for contact tracing purposes					
10 Discuss services that might help the patient to successfully isolate or quarantine at home					

Please provide your clinical care setting:

1. Outpatient clinic
2. Doctors office
3. Federally qualified health center
4. Hospital
5. Non‐healthcare
6. Pharmacy
7. Rural health center
8. Urgent care
9. Local health department
10. Other

Respondents were provided with these websites at the end of the survey:

- https://www.cms.gov/files/document/covid‐provider‐patient‐counseling‐checklist.pdf
- https://www.cms.gov/files/document/covid‐provider‐counseling‐qa.pdf
- https://www.cms.gov/files/document/covid‐provider‐patient‐counseling‐talking‐points.pdf
- https://www.cdc.gov/coronavirus/2019‐ncov/downloads/php/318271‐A_FS_KeyStepsWhenWaitingForCOVID‐19Results_3.pdf
